# Psychometric evaluation of the Symptoms of Infection with Coronavirus-19 (SIC): results from a cross-sectional study and a phase 3 clinical trial

**DOI:** 10.1186/s41687-023-00581-z

**Published:** 2023-05-17

**Authors:** Eric K.H. Chan, Valerie Williams, Carla Romano, Sheri Fehnel, Ashley F. Slagle, Jeffrey Stoddard, Jerald Sadoff, Margaret Mayorga, Sandy Lewis, Stuart Yarr, Jia Ma, Yan Liu, Eva G. Katz, Pauline McNulty, Ilse van Dromme, Kelly McQuarrie

**Affiliations:** 1 Janssen Global Services, LLC, Raritan, NJ USA; 2 RTI Health Solutions, Research Triangle Park, NC USA; 3 Aspen Consulting, LLC, Steamboat Springs, CO USA; 4 Janssen Research & Development, LLC, Raritan, NJ USA; 5 Janssen Research & Development, Beerse, Belgium; 6 Janssen Global Services, LLC, Horsham, PA USA

**Keywords:** Patient-reported outcome, COVID-19, Reliability, Validity, Psychometric evaluation

## Abstract

**Background:**

The Symptoms of Infection with Coronavirus-19 (SIC) is a 30-item patient-reported outcome (PRO) measure scored by body system composites to assess signs/symptoms of coronavirus disease 2019 (COVID-19). In addition to cross-sectional and longitudinal psychometric evaluations, qualitative exit interviews were conducted to support the content validity of the SIC.

**Methods:**

In a cross-sectional study, adults diagnosed with COVID-19 in the United States completed the web-based SIC and additional PRO measures. A subset was invited to participate in phone-based exit interviews. Longitudinal psychometric properties were assessed in ENSEMBLE2, a multinational, randomized, double-blind, placebo-controlled, phase 3 trial of the Ad26.COV2.S COVID-19 vaccine. Psychometric properties evaluated included structure, scoring, reliability, construct validity, discriminating ability, responsiveness, and meaningful change thresholds of SIC items and composite scores.

**Results:**

In the cross-sectional study, 152 participants completed the SIC (mean age, 51.0 ± 18.6 years) and 20 completed follow-up interviews. Fatigue (77.6%), feeling unwell (65.8%), and cough (60.5%) were symptoms most frequently reported. SIC inter-item correlations were all positive and mostly moderate (r ≥ 0.3) and statistically significant. SIC items and Patient-Reported Outcomes Measurement Information System-29 (PROMIS-29) scores correlated as hypothesized (all r ≥ 0.32). Internal consistency reliabilities of all SIC composite scores were satisfactory (Cronbach’s alpha, 0.69–0.91). SIC composite scores correlated moderately (r = 0.30–0.49) to strongly (r ≥ 0.50) with PROMIS-29 scores and Patient Global Impression of Severity (PGIS) ratings (all *P* < 0.01). A variety of signs/symptoms were cited in exit interviews, and participants considered the SIC straightforward, comprehensive, and easy to use. From ENSEMBLE2, 183 participants with laboratory-confirmed moderate to severe/critical COVID-19 were included (51.5 ± 14.8 years). Strong test-retest reliabilities were observed for most SIC composite scores (intraclass correlations ≥ 0.60). Statistically significant differences across PGIS severity levels were found for all but 1 composite score, supporting known-groups validity. All SIC composite scores demonstrated responsiveness based on changes in PGIS.

**Conclusions:**

The psychometric evaluations provided strong evidence for the reliability and validity of the SIC for measuring COVID-19 symptoms, supporting its use in vaccine and treatment trials. In exit interviews, participants described a broad range of signs/symptoms consistent with previous research, further supporting the content validity and format of the SIC.

**Supplementary Information:**

The online version contains supplementary material available at 10.1186/s41687-023-00581-z.

## Abstract (summary)

Coronavirus disease 2019 (COVID-19) is a serious disease that continues to evolve globally. Researchers developed the Symptoms of Infection with Coronavirus-19 (SIC), a 30-item questionnaire designed for patients to report signs and symptoms of COVID-19. In this study, the researchers formally analyzed how well the SIC measures the patient experience with COVID-19, using survey and clinical trial data as well as telephone interviews. Adults with COVID-19 and at least 2 bothersome symptoms completed the web-based survey, and some of these individuals also participated in in-depth interviews. Participants in a clinical trial for a COVID-19 vaccine also completed the SIC measure. The SIC was compared with other commonly used questionnaires that evaluate patient experience. The most commonly reported symptoms of COVID-19 were fatigue, feeling unwell, cough, weakness, and headache. The items for individual symptoms (e.g., “cough”) and combined scores for body systems (e.g., “respiratory system”) performed well in statistical analyses. Participants found the SIC to be straightforward, comprehensive, and easy to use. The SIC may prove useful in the future for vaccine and treatment trials for COVID-19.

**Supplementary Information:**

The online version contains supplementary material available at 10.1186/s41687-023-00581-z.

## Background

According to the Centers for Disease Control and Prevention (CDC), as of September 2022, there were > 95 million total cases of coronavirus disease 2019 (COVID-19) in the United States and 1 million deaths [1]. As of September 2022, 68% of the eligible US population was fully vaccinated, including 92% of individuals aged ≥ 65 years and 72% of the population aged ≥ 5 years [1]. Booster doses lag behind, with only 49% of eligible people having received 1 booster dose. Worldwide, COVID-19 has resulted in 610 million cases and caused > 6.5 million deaths [2]. The emergence of new and increasingly transmissible variants [3–5] highlights the continued need for both vaccination and characterization of COVID-19 signs and symptoms as part of the global effort against the pandemic.

Some of the symptoms associated with COVID-19 include fever or chills, dry cough, shortness of breath, fatigue, muscle or body aches, headache, loss of taste or smell, sore throat, congestion or runny nose, red eyes, skin rash, discoloration of fingers or toes, nausea or vomiting, and diarrhea [6, 7]. Some of these symptoms can resemble those of other respiratory diseases, particularly influenza [6, 8]. Certain distinct symptoms, such as sudden loss of smell and taste, may indicate COVID-19 infection [9, 10]. Some populations are at increased risk of severe COVID-19, with key risk factors including age ≥ 65 years; male sex; and comorbidities such as heart disease, diabetes, immunocompromised states, or chronic lung, liver, or kidney disease.

The patient experience with COVID-19 varies across individuals and differs from other viral infections in terms of types of symptoms and their severity, with patients experiencing a range of respiratory, neurologic, gastrointestinal, circulatory, and musculoskeletal symptoms [6, 9, 11]. In addition to the variety of acute symptoms, many patients experience long-term symptoms [12]. Prospective studies of signs and symptoms of COVID-19 are needed to characterize disease presentation and progression, especially in light of new variants, and to evaluate diagnostic tests, treatments, and vaccines [9]. Patient-reported outcome (PRO) instruments provide a standardized method to assess the disease experience and impact from the patient perspective, and can be used to monitor disease progression and response to treatment [13, 14]. In the context of COVID-19, PRO measures may facilitate diagnosis, contact tracing and prevention strategies, long-term follow-up, and may inform clinical trial design [13]. COVID-19–specific PRO measures are needed to help accurately measure COVID-19 signs and symptoms and capture changes in disease severity.

At the time of this study, no COVID-19–specific PRO measures were published, and few have been developed to date [13]. PRO instruments developed for the diagnosis of COVID-19 include the Italian EPICOVID19 screen for 11 patient-reported symptoms [15] and the UK COVID-19 Symptom Study app based on 1 to 3 days of self-reported symptoms (among a total of 19 possible symptoms) [16]. The US CDC Coronavirus Self-Checker is an online tool that offers recommendations based on the user’s responses [17]. The US Occupational Safety and Health Administration employee COVID-19 health screening questionnaire includes 11 self-reported symptoms [18]. Other health surveys have been developed by workplaces, universities, laboratories, federal agencies, and other entities [19]. Many of the prominent symptoms reported in the SIC, such as fatigue and cough, have been reported in other PRO studies [11]. Newer instruments assess health-related quality of life in current/former COVID-19 patients [20] and/or post–acute sequelae of SARS-CoV-2 infection (“long COVID”) [21]. The SIC is unique in that it is a PRO measure designed to capture presence and severity of acute COVID-19 symptoms, rather than serving as an epidemiological or surveillance tool.

The purpose of this study was to perform a psychometric evaluation of the Symptoms of Infection with Coronavirus-19 (SIC), a PRO measure designed to assess the presence and severity of signs and symptoms of COVID-19. To be appropriate for use in both vaccine and treatment clinical studies, we sought to create a measure that was sensitive to both disease onset and changes in key signs and symptoms of COVID-19 using best practices in alignment with current regulatory guidance [22–26].

## Methods

### Description of the SIC

**Fig. 1:**
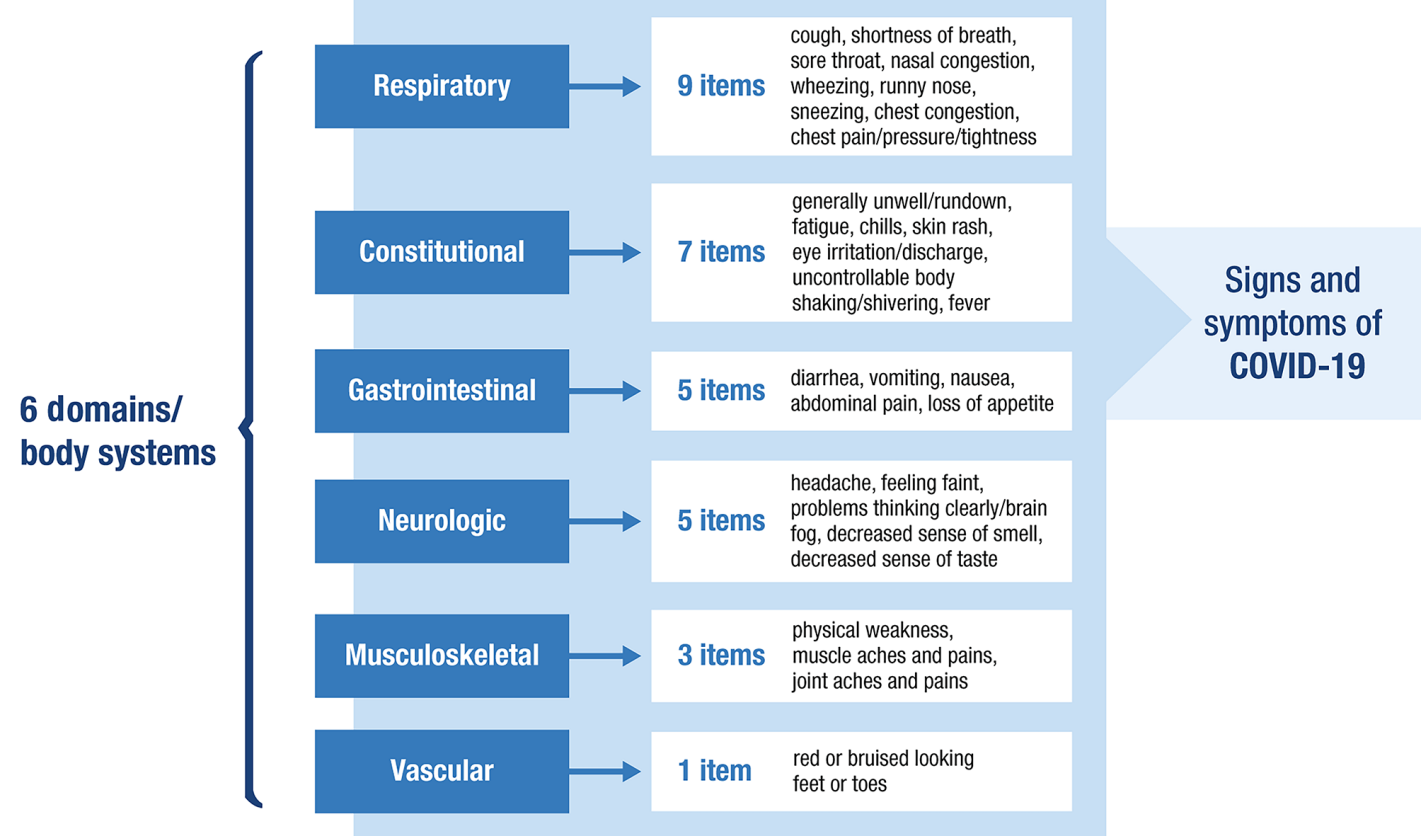
**SIC conceptual framework** [27]**.** SIC, Symptoms of Infection with Coronavirus-19; COVID-19, coronavirus disease 2019

The SIC includes 30 sign/symptom items grouped into composite scores by body system, including respiratory (9 items), constitutional (7 items), gastrointestinal (5 items), neurologic (5 items), musculoskeletal (3 items), and vascular (1 item) (Fig. 1) [27]. Participants rate the signs and symptoms as present or absent (yes or no) during the past 24 h. Of the 30 signs and symptoms, 25 are rated on a severity scale of 0 (none) through 10 (worst possible), while 5 additional items are rated only as present or absent (Table S1). The SIC composite scores are constructed as the average of the symptom severity ratings for each set of items. The development and content validation of the SIC were based on a targeted review of the literature, interviews with patients with COVID-19, experts in infectious diseases, and clinicians who treat patients with COVID-19 (Figure S1). In the initial development of the SIC, 3 iterative rounds of combined concept elicitation and cognitive debriefing interviews were conducted with a total of 31 patients, caregivers, and healthy volunteers. Using data collected from a cross-sectional observational study, we examined the measurement properties of each SIC item, as well as composite scores based on these items. In the cross-sectional study, qualitative exit interviews were conducted in a subgroup of participants to strengthen the content validity of the SIC. Psychometric properties of the SIC, including test-retest reliability, known-groups validity, responsiveness, and meaningful change thresholds of composite scores, were also assessed using a subset of data from a phase 3 trial of the Ad26.COV2.S COVID-19 vaccine (ENSEMBLE2; ClinicalTrials.gov Identifier: NCT04614948) [28].

### Cross-sectional study design

In the cross-sectional, observational study, SIC data were collected via a web-based survey from US adults with COVID-19 based on a positive test and current symptoms. Inclusion criteria were the following: adults (aged ≥ 18 years) with a self-reported positive COVID-19 test within 2 weeks of screening; ≥ 2 current, bothersome symptoms of COVID-19; and willingness to provide medical and demographic information and to complete the survey. Recruitment targets for the survey were set to ensure a diverse sample for age (up to 50% < 65 years old), hospitalization status (≥ 35 patients hospitalized because of COVID-19), preexisting comorbidities (causing higher risk for severe disease), race/ethnicity (up to 50% non-White), and disease severity (mild, moderate, and severe). Recruitment targets for the exit interviews also aimed for a diverse population with ≥ 50% minority status, with highest priority given to race/ethnicity other than White, education level less than high school/General Educational Development and variable access to health care. Participants received 100 USD in appreciation of their time for completing the web-based survey and an additional 100 USD for completion of the exit interview.

Participants in the cross-sectional study completed the SIC, a study-specific Overall Symptom Checklist for the 30 SIC items (to identify symptoms that had been experienced but resolved; yes or no for each item), and an open-ended response option for any symptoms they experienced that were not captured in the checklist. Participants were asked 2 additional questions about what symptoms had to improve or resolve before they felt they could return to their usual activities and 4 questions about their access to and receipt of medical care related to COVID-19 (Table S2).

Psychometric performance of the SIC was evaluated using other PRO measures, including the Patient Global Impression of Severity (PGIS) [29, 30], Patient Global Impression of Change (PGIC) [29], Patient-Reported Outcomes Measurement Information System-29 (PROMIS-29) [31], and the Overall Symptom Checklist. The PGIS is a single-item, patient-reported, 4-point rating scale that assesses the severity of COVID-19 symptoms over a 24-h period as follows: 0 = none, 1 = mild, 2 = moderate, and 3 = severe. The PGIC is a single-item, patient-reported, 7-point rating scale that evaluates change in severity of COVID-19 symptoms since onset as follows: 0 = much better, 1 = moderately better, 2 = a little better, 3 = no change, 4 = a little worse, 5 = moderately worse, and 6 = much worse. The PROMIS-29 is a PRO measure that assesses 7 domains: depression, anxiety, physical function, pain interference, fatigue, sleep disturbance, and ability to participate in social roles and activities. There are 29 items in total, with all 7 domains including 4 items each (rated from 1 to 5), plus an item assessing pain intensity (rated from 0 to 10). Higher scores indicate more of the concept being measured (i.e., more depression or more physical function).

The EQ-5D-5L was used on an exploratory basis to rate current health status. The EQ-5D-5L evaluates 5 dimensions: mobility, daily activities, self-care, pain/discomfort, and anxiety/depression [32]. Dimensions are rated as: 1 = no problems, 2 = slight problems, 3 = moderate problems, 4 = severe problems, and 5 = extreme problems. Global health is also measured on a visual analog scale from 0 (worst imaginable health) to 100 (best imaginable health).

After completing the web-based survey, participants were asked to participate in an in-depth exit interview using a semistructured guide to provide further details regarding their COVID-19 experience. Analysis of the exit interview data used a thematic qualitative approach with information from field notes and transcripts to identify dominant trends and generate themes or patterns in respondents’ symptom reports. Common themes were identified via constant comparative analysis, which also allowed confirmation of content validity and patient comprehension of the SIC.

### Phase 3 trial study design

ENSEMBLE2 was a multinational, randomized, double-blind, placebo-controlled, phase 3 trial assessing the safety and efficacy of the Ad26.COV2.S vaccine for the prevention of COVID-19 in adults [28]. Participants from Europe, the United States, Latin America, South Africa, and the Philippines were randomized 1:1 to receive a primary dose of Ad26.COV2.S or placebo, plus a homologous booster dose or placebo dose at a 2-month interval. The study consisted of a 60-week study period and a follow-up period of 1 year. Eligible participants were adults (aged ≥ 18 years), who were healthy or with stable and well-controlled comorbidities, without prior receipt of a COVID-19 vaccine. Additional inclusion criteria for the current study of the SIC used in ENSEMBLE2 were: polymerase chain reaction (PCR)–confirmed COVID-19 from a central laboratory (University of Washington); COVID-19 occurring from Days 15 to 56 (prior to booster vaccination); moderate to severe/critical COVID-19, assessed by a blinded Clinical Severity Adjudication Committee; and SIC data collected within 7 days of PCR confirmation. ENSEMBLE2 recruitment targeted a diverse representation of race/ethnicity and sex.

In the ENSEMBLE2 trial, 31,300 participants were enrolled and randomized to receive Ad26.COV2.S (n = 15,708) or placebo (n = 15,592); a subset of participants was included in the present analysis. All participants in the ENSEMBLE2 trial were provided an electronic clinical outcome assessment tool, completed the SIC prior to first vaccine administration, and were monitored for the development of COVID-19 symptoms throughout the study. Participants reporting symptoms that suggested possible COVID-19 and those reporting any non-study positive-PCR tests were asked to complete 2 PRO measures daily throughout each suspected COVID-19 episode: the SIC was used to evaluate COVID-19 signs and symptoms, and the PGIS was used for validation. Additionally, participants reported blood oxygen saturation and pulse rate 3 times per day and body temperature daily during a COVID-19 episode. Nasal swabs were collected for detection of SARS-CoV-2 infection by reverse transcription (RT)–PCR within 1 to 2 days of symptom onset, on Days 3 to 5, and then every 2 days. Participants with ≥ 1 positive SARS-CoV-2 nasal swab returned to the study site 28 days (± 7 days) after symptom onset for clinical assessment and seroconfirmation of SARS-CoV-2 infection. Resolution of a COVID-19 episode was established by 2 consecutive, negative, SARS-CoV-2 nasal swabs and 2 consecutive days without COVID-19–related signs or symptoms.

### Psychometric evaluation

Psychometric analyses were conducted using data from participants who completed the SIC in the cross-sectional study and a subset of participants from ENSEMBLE2. The psychometric properties evaluated in each study for all SIC items and candidate composite scores are summarized in Table 1. All statistical tests were 2-tailed (α = 0.05), and *P* ≤ 0.05 was considered statistically significant.

**Table 1:** Psychometric Properties Assessed in the Cross-sectional Study and ENSEMBLE2 Phase 3 Trial

Psychometric analyses	Cross-sectional study	ENSEMBLE2
Item-level descriptive statistics	X	
Inter-item correlations	X	
Item-level construct validity	X	
Item-level known-groups (discriminating ability)	X	
Test-retest reliability		X
Scoring	Preliminary	Confirmatory
Internal consistency reliability	X	X
Composite-level descriptive statistics	X	
Composite-level construct validity	X	
Composite-level known-groups (discriminating ability)	X	X
Composite-level ability to detect change (responsiveness)		X
Meaningful change thresholds (based on global severity item)		X

Descriptive statistics for the SIC were computed in the cross-sectional study for COVID-19 signs and symptoms, including for subgroups based on age (< 65 or ≥ 65 years) and comorbidities (0 vs. ≥ 1); response frequency distributions were calculated for each SIC item. Inter-item correlations for the SIC items were computed and examined for expected patterns of relationships.

Construct validity of the SIC was assessed in the cross-sectional study by correlational analyses, with the strength and sign of the predicted correlation depending on the expected pattern of relationship for measures addressing similar constructs. SIC fatigue scores were hypothesized to correlate at least moderately with PROMIS-29 fatigue scores, SIC musculoskeletal items to correlate at least moderately with PROMIS-29 physical function scores, and SIC pain items to correlate at least moderately with PROMIS-29 pain scores. Correlation coefficients were interpreted as follows: r ≥ 0.50, large; 0.30 to 0.49, moderate; 0.10 to 0.29, small; and < 0.10, trivial.

Known-groups validity (distinguishing among hypothetically different groups) was evaluated in the cross-sectional study and in ENSEMBLE2 via analysis of variance (ANOVA) to compare mean differences in SIC item scores between subgroups: participants reporting no symptoms or mild compared to those reporting moderate or severe on the PGIS; participants with scores in the top one-third of the PROMIS-29 physical function score distribution versus participants in the bottom one-third; and respondents with PROMIS-29 fatigue scores in the top one-third of the distribution versus those in the bottom one-third.

A set of candidate SIC composite scores representing different body systems was analyzed in both studies. Candidate scoring algorithms were evaluated with respect to internal consistency reliability and composite-level analyses that paralleled the item-level validity analyses. Although not ideal for the evaluation of symptom measures such as the SIC, Cronbach’s coefficient alpha was calculated to explore the internal consistency of the candidate SIC composite scores.

In the ENSEMBLE2 trial, test-retest reliability (reproduction of identical measurements when no change has occurred between testing) was assessed by calculating intraclass correlation coefficients in the subgroup of participants identified as stable by PGIS response (“no symptom,” “mild,” or same response) at Day 1 and Day 2 of their COVID-19 episode [33]. The intraclass correlation was estimated using a mixed effects model, calculated as estimated within-subject variance over estimated total variance. The responsiveness of the SIC was evaluated in ENSEMBLE2 using the PGIS as an anchor variable via change in SIC composite scores from Days 1 to 2 (ANOVA) to assess the ability to detect changes in symptoms over time. Meaningful change thresholds (i.e., the smallest change that patients perceive as beneficial) of the SIC were estimated in ENSEMBLE2 using ANOVAs and mean change scores (1- or 2-point improvement in PGIS).

### Ethics

All participants provided informed consent. The cross-sectional study was reviewed on ethical grounds by the RTI International institutional review board. The ENSEMBLE2 trial adhered to the International Council for Harmonisation guidelines on Good Clinical Practice and the principles of the Declaration of Helsinki.

## Results

### Demographics

In the cross-sectional study, 152 participants completed the PRO measures from November 17, 2020, to January 4, 2021 (mean ± standard deviation [SD] age, 51.0 ± 18.6 years [range, 18–90 years]; female, 62.5%; Table 2). Respondents were racially and ethnically diverse, and approximately half reported ≥ 1 comorbid condition. Most participants reported their worst symptoms as moderate (47.4%) versus mild (26.3%) or severe (26.3%); patients had experienced symptoms for a mean duration of 14.9 ± 7.3 days. Among those who had been hospitalized (27.6%), the mean stay was 3.2 ± 2.4 days.

**Table 2:** Participant Characteristics at Screening (Cross-sectional Study)

Characteristic	Cross-sectional study (N = 152)
Age, years, mean (SD)	51.0 (18.6)
Median (range)	53.5 (18.0–90.0)
Sex, n (%)	
Male	57 (37.5)
Female	95 (62.5)
Race, n (%)	
White	87 (57.2)
African American or Black	38 (25.0)
American Indian	2 (1.3)
Asian	2 (1.3)
Mixed race (2 or more races)	16 (10.5)
Other	6 (3.9)
Prefer not to answer	1 (0.7)
Hispanic or Latino	30 (19.7)
Highest level of education, n (%)	
Less than high school	4 (2.6)
High school or GED	24 (15.8)
Some college	35 (23.0)
Associate degree	20 (13.2)
College degree	45 (29.6)
Postgraduate degree	24 (15.8)
Smoking or tobacco use (self or family members), n (%)	
Yes	27 (17.8)
No	125 (82.2)
Diagnosed comorbidities, n (%)	
Hypertension	38 (25.0)
Type 1 diabetes	4 (2.6)
Heart disease	18 (11.8)
Chronic obstructive pulmonary disease	10 (6.6)
Asthma	24 (15.8)
Obesity	29 (19.1)
None of the above	79 (52.0)

SD, standard deviation; GED, General Educational Development

Twenty of the 152 participants who completed the SIC also completed follow-up interviews from January 7 to February 17, 2021 (mean ± SD age, 51.5 ± 16.8 years [range, 22–73 years]; female, 65%). Interview participants were racially diverse, with 45.0% African American, 25.0% White, 5.0% American Indian, 10.0% mixed race, and 15.0% other race, and had a range of educational levels, employment statuses, and types of health insurance coverage. Approximately half of the exit interview participants (45.0%; 9/20) had a preexisting risk factor increasing the likelihood of severe COVID-19 outcomes. Preexisting conditions included asthma (45.0%), obesity (35.0%), hypertension (35.0%), heart disease (25.0%), chronic obstructive pulmonary disease (10.0%), and type 1 diabetes (5.0%); some participants reported > 1 condition. COVID-19 was reported as severe in 45.0%, moderate in 35.0%, and mild in 20.0% of interview participants, and 45.0% (9/20) had been hospitalized for COVID-19; of those, 4 had received oxygen and 2 were admitted to the intensive care unit.

From ENSEMBLE2 (November 16, 2020, to June 25, 2021), 183 participants with confirmed moderate to severe/critical COVID-19 occurring from Days 15 to 56 post–primary vaccination, but before planned booster vaccination, were included in this analysis (mean ± SD age, 51.5 ± 14.8 years [range, 18–85]; female, 43.7%). Participants were 65.0% White, 18.0% Asian, 13.7% American Indian or Alaska Native, and 2.7% Black. Participants came from Europe (32.2%), the United States (27.9%), Latin America (23.0%), the Philippines (13.1%), and South Africa (3.8%). Of these 183 participants, 130 completed the SIC.

### Psychometric analyses

#### Item-level descriptive statistics

The most frequently endorsed SIC symptoms in the cross-sectional study were fatigue (77.6%), feeling unwell (65.8%), cough (60.5%), physical weakness (59.9%), and headache (59.9%) (Table 3). Mean overall severity scores on the scale of 0 to 10 ranged from 2.95 for cough to 4.68 for fatigue. For participants aged < 65 years (n = 88), fatigue had the highest severity rating (mean, 4.59) followed by headache (3.78), feeling unwell (3.43), and physical weakness (3.36). For participants aged ≥ 65 years (n = 64), fatigue (4.80) had the highest severity rating followed by feeling unwell (4.23), loss of appetite (3.61), and physical weakness (3.44). In comorbidity subgroups, grouped as 0 (n = 79) versus ≥ 1 (n = 73), fatigue had the worst severity scores in both groups (4.70 for no comorbidities vs. 4.66 for those with comorbidities) followed by feeling unwell (3.82 vs. 3.71); participants without comorbidities generally had lower mean scores on the SIC items. In ENSEMBLE2, the most frequently endorsed SIC symptoms were similar to those reported in the cross-sectional study and included feeling unwell, cough, headache, fatigue, and sore throat (Table S3). Most respondents reported their overall symptoms as mild (n = 66, 43.4%) or moderate (n = 54, 35.5%) on the PGIS and rated their change in symptoms since symptom onset as much better (n = 56, 36.8%) or moderately better (n = 40, 26.3%) on the PGIC (Table S4).

**Table 3:** Frequently Endorsed SIC Item-level Responses and Severity Score (Cross-sectional Study)

	Symptom frequency, n (%; N = 152)		SIC severity score, mean (SD)^a^		
**SIC item**	No	Yes	Age < 65 years (n = 88)	Age ≥ 65 years (n = 64)	Overall (n = 152)
Fatigue	34 (22.4)	118 (77.6)	4.59 (3.2)	4.80 (3.0)	4.68 (3.1)
Feeling unwell	52 (34.2)	100 (65.8)	3.43 (3.2)	4.23 (3.1)	3.77 (3.2)
Cough	60 (39.5)	92 (60.5)	2.92 (2.9)	3.00 (3.2)	2.95 (3.0)
Physical weakness	61 (40.1)	91 (59.9)	3.36 (3.3)	3.44 (3.3)	3.39 (3.3)
Headache	61 (40.1)	91 (59.9)	3.78 (3.5)	2.83 (3.0)	3.38 (3.3)

SIC, Symptoms of Infection with Coronavirus-19; SD, standard deviation.

^a^SIC item scores range from 0 (not experienced or none) to 10 (worst possible), with higher values reflecting worse symptom severity.

#### Item-level analyses

Results from the cross-sectional study showed that all SIC inter-item correlations were positive in sign and correlations within each composite score were generally statistically significant and at least moderate in size (r ≥ 0.30).

In the cross-sectional study, construct validity correlations between SIC items and PROMIS-29 scores followed the hypothesized patterns. As expected, the SIC fatigue score correlated positively with PROMIS-29 fatigue score (r = 0.64) and negatively with the physical function score (r = − 0.65); SIC physical weakness, muscle aches/pains, and joint aches/pains scores correlated moderately to strongly with PROMIS-29 physical function scores (r = − 0.67, r = − 0.52, and r = − 0.42, respectively); and the 5 SIC pain items correlated at least moderately with PROMIS-29 pain scores (all r ≥ 0.32).

Respondents with mild or no symptoms on the PGIS had better item-level SIC scores compared with the moderate or severe PGIS subgroups. Most differences between PGIS subgroups were statistically significant, with the exception of the chest congestion, eye irritation, abdominal pain, and red/bruised feet items. Respondents with better PROMIS-29 physical function scores and better PROMIS-29 fatigue scores also had better SIC scores, with statistically significant differences observed except for the vomiting item (with PROMIS-29 physical function) and skin rash, uncontrollable shaking/shivering, and red/bruised feet (with PROMIS-29 fatigue). As expected, the largest mean difference between PROMIS-29 physical function subgroups was for the SIC physical weakness item and the largest difference between PROMIS-29 fatigue subgroups was for the SIC fatigue and physical weakness items. These item-level known-group analyses provided strong support for the discriminating ability of the SIC items.

#### Composite-level analyses

SIC composite scores were formed by body system and included constitutional (7 items), gastrointestinal (5 items), musculoskeletal (3 items), neurologic (3 items), sensory (2 items), and respiratory (9 items) composites. A single vascular item was scored on its own (0 = no and 1 = yes). SIC scores were also computed separately for upper and lower respiratory symptoms.

Internal consistency reliabilities indicated that most of the SIC composite scores comprised items that were strongly related, with Cronbach’s alpha > 0.70 for all but 1 SIC composite score in either the cross-sectional study (gastrointestinal) and ENSEMBLE2 (neurologic) (Table 4).

**Table 4:** SIC Internal Consistency Reliabilities (Cross-sectional Study, N = 152; ENSEMBLE2 Trial, N = 130^a^)

SIC composite score	Cronbach’s alpha,^b^ cross-sectional study	Cronbach’s alpha,^b^ ENSEMBLE2^c^	No. of items
Constitutional	0.72	0.71	6
Gastrointestinal	0.69	0.71	5
Musculoskeletal	0.86	0.82	3
Neurologic	0.72	0.41	3
Sensory	0.91	0.87	2
Respiratory	0.87	0.82	9
Upper respiratory	0.80	0.80	4
Lower respiratory	0.84	0.75	5

COVID-19, coronavirus disease 2019; SIC, Symptoms of Infection with Coronavirus-19; PCR, polymerase chain reaction.

^a^Of 183 participants with PCR-confirmed moderate to severe/critical COVID-19 occurring from Days 15 to 56 post–primary vaccination, 130 completed the SIC.

^b^Cronbach’s alpha ≥ 0.70 indicates a set of items that is strongly related.

^c^SIC completed at Day 1 of a COVID-19 episode in ENSEMBLE2.

In the cross-sectional study, SIC composite scores correlated moderately (r = 0.30–0.49) to strongly (r ≥ 0.50) with PROMIS-29 scores, and all were statistically significant (construct validity; Table 5). Further, most EQ-5D-5L scores correlated moderately to strongly with SIC composite scores; EQ-5D-5L scores for pain/discomfort, self-care, and usual activities correlated most strongly with SIC constitutional and musculoskeletal scores (r = 0.58–0.65; Table 6). SIC composite scores correlated moderately to strongly and positively with the PGIS, with correlations ranging from 0.41 for the upper respiratory score to 0.73 for the constitutional score (Table 6).

**Table 5:** SIC Construct Validity Correlations With PROMIS-29 Scores (Cross-sectional Study, N = 152)

	PROMIS-29 scores							
SIC composite score	Depression	Anxiety	Physical function	Pain interference	Fatigue	Sleep disturbance	Social activities	Pain intensity
Constitutional	0.38	0.36	− 0.59	0.44	0.52	0.41	− 0.55	0.52
Gastrointestinal	0.39	0.35	− 0.47	0.47	0.43	0.31	− 0.54	0.51
Musculoskeletal	0.48	0.36	− 0.61	0.56	0.56	0.45	− 0.59	0.61
Neurologic	0.36	0.37	− 0.47	0.48	0.44	0.35	− 0.45	0.52
Sensory	0.37	0.42	− 0.50	0.25	0.48	0.30	− 0.42	0.29
Respiratory	0.42	0.28	− 0.47	0.43	0.41	0.30	− 0.48	0.50
Upper respiratory	0.34	0.19	− 0.30	0.34	0.27	0.21	− 0.33	0.39
Lower respiratory	0.39	0.30	− 0.52	0.42	0.44	0.31	− 0.50	0.48

SIC, Symptoms of Infection with Coronavirus-19; PROMIS-29, Patient-Reported Outcomes Measurement Information System-29

All *P* < 0.05 for H_0_: ρ = 0

**Table 6:** SIC Correlations With the PGIS and EQ-5D-5L (Cross-sectional Study, N = 152)

**SIC composite score**	PGIS	EQ-5D-5L					
		Mobility	Usual activities	Self-care	Pain/discomfort	Anxiety	VAS
Constitutional	0.73	0.46	0.58	0.58	0.61	0.43	− 0.56
Gastrointestinal	0.55	0.36	0.45	0.41	0.53	0.46	− 0.41
Musculoskeletal	0.66	0.53	0.59	0.63	0.65	0.53	− 0.55
Neurologic	0.52	0.33	0.57	0.40	0.57	0.46	− 0.51
Sensory	0.47	0.35	0.42	0.35	0.36	0.36	− 0.36
Respiratory	0.58	0.44	0.46	0.48	0.51	0.45	− 0.35
Upper respiratory	0.41	0.25	0.27	0.29	0.31	0.33	− 0.18
Lower respiratory	0.58	0.49	0.51	0.53	0.55	0.45	− 0.41

SIC, Symptoms of Infection With Coronavirus-19; PGIS, Patient Global Impression of Severity; VAS, visual analog scale

All *P* < 0.05 for H_0_: ρ = 0

**Fig. 2:**
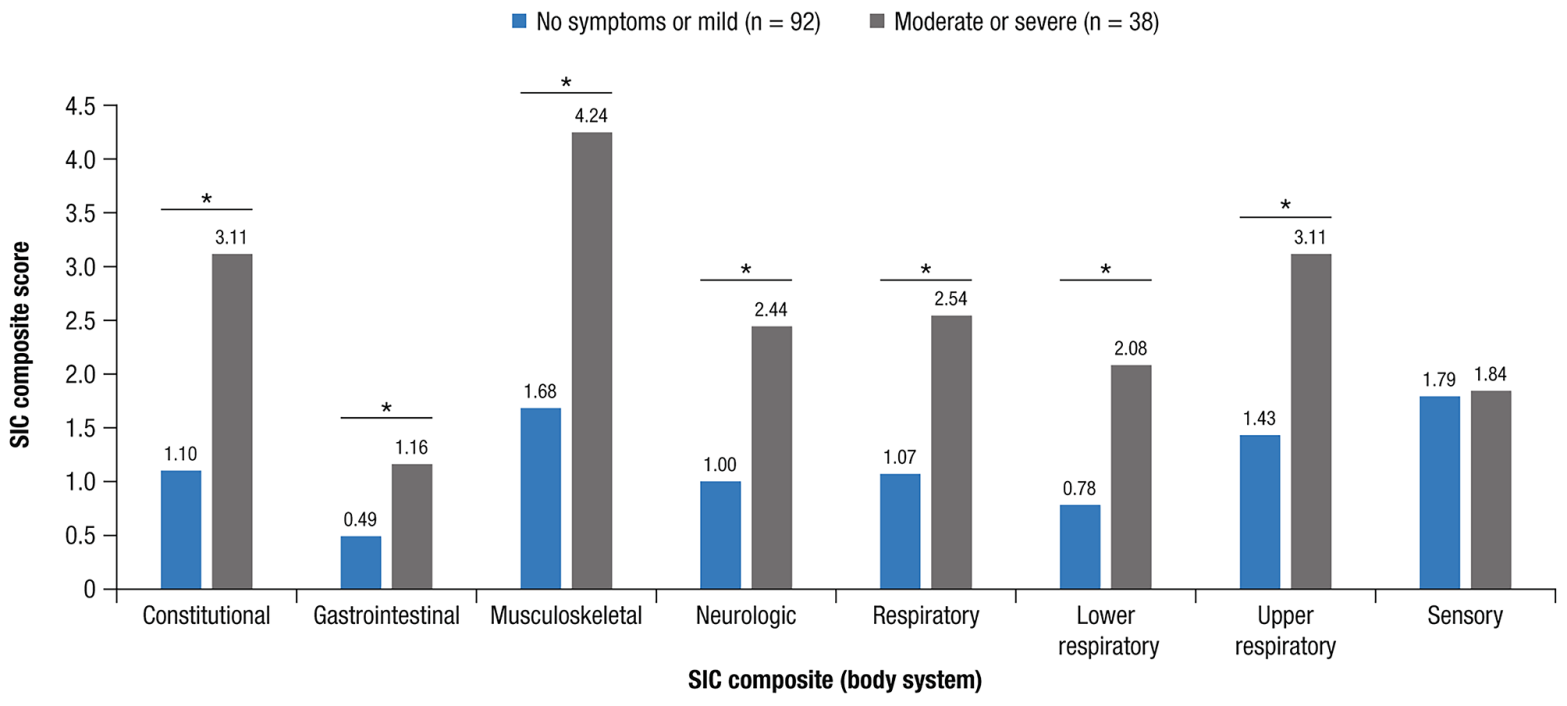
**SIC composite score known-group validity by Day 1 PGIS response (ENSEMBLE2 trial; N = 130**^**a**^**).** SIC, Symptoms of Infection with Coronavirus-19; PGIS, Patient Global Impression of Severity; PCR, polymerase chain reaction; COVID-19, coronavirus disease 2019. ^a^Of 183 participants with PCR-confirmed moderate to severe/critical COVID-19 occurring from Days 15 to 56 post–primary vaccination, 130 completed the SIC. *P < 0.05

In the cross-sectional study, the known-groups analyses showed that all composite-level differences were in the expected direction and all were statistically significant. The no symptoms/mild PGIS subgroup achieved better SIC scores than the moderate/severe PGIS subgroup. Respondents in the top one-third of the distribution of PROMIS-29 physical function scores achieved better SIC scores than those in the bottom one-third, and respondents in the top one-third of the distribution of PROMIS-29 fatigue scores had better SIC scores than the bottom one-third. In ENSEMBLE2, statistically significant differences (*P* < 0.05) were observed for the composite scores for the different PGIS severity levels except for the sensory composite (Fig. 2), supporting known-groups validity.

**Fig. 3:**
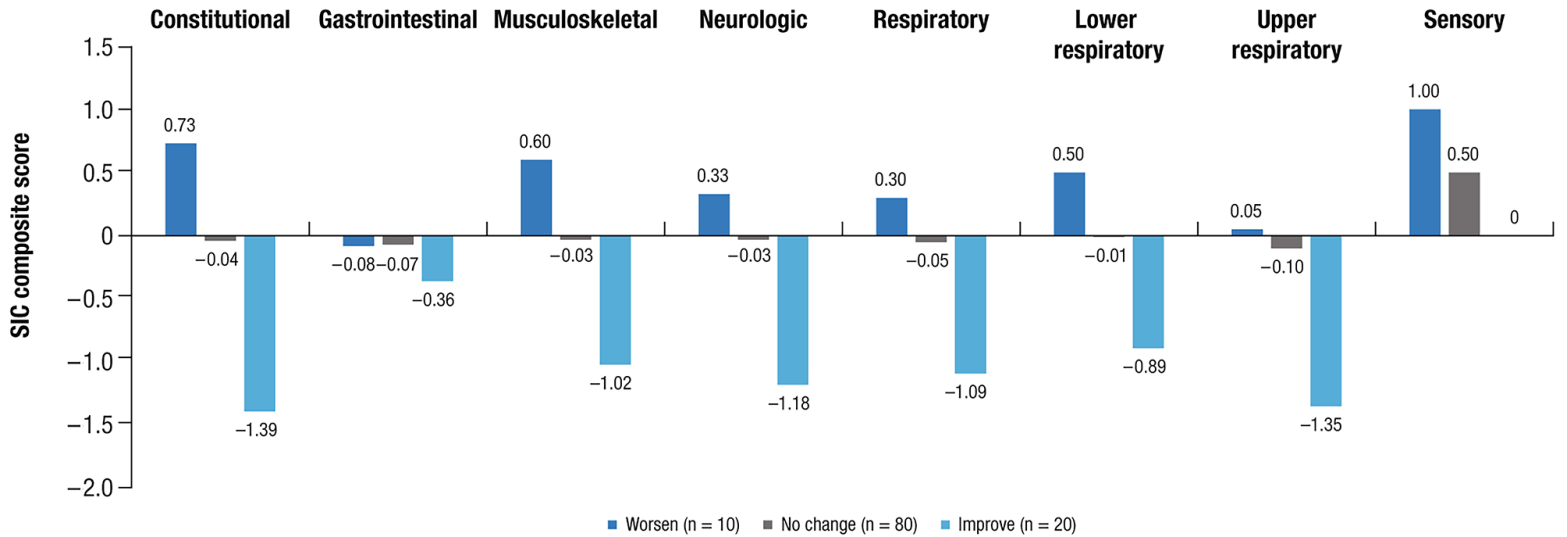
**SIC composite score**^a^ **change by Day 2 PGIS status (ENSEMBLE2 trial; N = 130**^**b**^**)**. SIC, Symptoms of Infection with Coronavirus-19; PGIS, Patient Global Impression of Severity; PCR, polymerase chain reaction; COVID-19, coronavirus disease 2019. ^a^Change in composite score is the composite score at Day 2 minus the composite score at Day 1. A score < 0 indicates improvement, a score of 0 indicates no change, and a score > 0 indicates worsening. ^b^Of 183 participants with PCR-confirmed moderate to severe/critical COVID-19 occurring from Days 15 to 56 post–primary vaccination, 130 completed the SIC.

In ENSEMBLE2, test-retest reliabilities were strong for most composite scores and were moderate for neurologic and constitutional (Table S5). The responsiveness of the SIC was apparent in all SIC composite scores, and improvement and worsening of PGIS ratings and SIC composite scores moved in the same direction, supporting the ability of the SIC to detect COVID-19 sign and symptom changes over time (Fig. 3). The estimated meaningful change thresholds for the SIC composite scores are based on 1- or 2-point improvements on the PGIS and ranged from − 0.21 for gastrointestinal at Day 3 to − 2.11 for musculoskeletal at Day 5 (Table S6).

### Exit interviews (cross-sectional study)

In exit interviews, participants reported a broad range of signs and symptoms of COVID-19; they did not describe a single, consistent constellation of symptoms. The top 7 symptoms reported among the 20 patients were fatigue (80.0%), cough and shortness of breath (both 65.0%), decreased sense of smell and taste (both 60.0%), headache (55.0%), and nasal congestion (50.0%). Loss of smell or taste prompted some participants to believe they had COVID-19 instead of a cold or flu. The progression and ultimate resolution of symptoms varied among patients and by the symptom in question. The most bothersome symptoms (identified as the greatest barriers preventing a return to usual activities) were shortness of breath (25.0%), fatigue (20.0%), and headache (15.0%). Most participants (85.0%) reported being negatively affected by needing to self-isolate from family and other people, and that they experienced anxiety regarding what would happen to them and family members. Participants seemed hesitant to label their COVID-19 experience as severe disease if they were not hospitalized or receiving supplemental oxygen, even though almost half (45.0%) reported symptoms consistent with severe disease. Table S7 presents representative quotations from the exit interviews.

Participants provided insight into meaningful change and recovery by describing which COVID-19 symptoms required complete resolution before resuming normal activities versus those that only required some improvement. Fatigue, shortness of breath, and vomiting were the top 3 symptoms participants described as needing to be resolved before they could resume activities. Other symptoms frequently cited as needing to be completely resolved were physical weakness, fever, chest pain, uncontrollable shivering, feeling generally unwell, diarrhea, and headache. Cough and congestion would need improvement, but not necessarily resolution, before resuming activities. Estimates of the degree of symptom improvement that could enable participants to return to everyday activities varied by symptom.

Respondents also answered 4 questions about health care access for their COVID-19 symptoms. Most respondents considered it “very easy” to access care (49.0%) and to get their most recent COVID-19 test (46.0%). More than half cited no barriers to receiving care (57.0%), although most did not seek medical care beyond COVID-19 testing (60.0%).

### Participants’ evaluation of the SIC (cross-sectional study)

When asked to review the format and content of the SIC, all participants perceived the SIC to be straightforward, comprehensive, and easy to use, even when they were moderately ill. Several participants indicated that self-report on the SIC would be challenging during severe illness or intubation, although measures could be (or were) taken with assistance from a caregiver or using fingers to indicate symptom severity. Overall, participants felt the SIC was an accurate measure to report and track COVID-19 symptom onset, emergence of new signs/symptoms, and changes in symptoms over time.

## Discussion

This study reports the psychometric properties of the SIC, a PRO measure with potential application in future vaccine and treatment trials for COVID-19. The results support the reliability and validity of the SIC items and composite scores as appropriate and useful measures of COVID-19 symptom severity. Results of the qualitative exit interviews in a diverse and potentially vulnerable population described a broad range of signs and symptoms of COVID-19 and were consistent with previous qualitative research, further supporting the content validity and format of the SIC. Exit interview participants further endorsed the SIC, noting that it was an accurate and easy method to describe COVID-19 symptom onset and changes over time. In the ENSEMBLE2 clinical trial, longitudinal psychometric data collected in a global population supported the test-retest reliability, known-groups validity, responsiveness, and meaningful change thresholds of the SIC composite scores.

Effective survey instruments for health care research should be easy and quick to administer, inexpensive, accessible, and convenient [34]. Exit interviews from the current study and an earlier qualitative study support the ease of use of the SIC by patients and caregivers and across varying disease severity [27]. The SIC was developed to align with current US Food and Drug Administration and patient-focused drug development guidance regarding the use of PRO measures in clinical trials and characterization of the patient experience of COVID-19. This guidance stipulates that any PRO measure used to collect patient experiences must be developed with extensive input from patients in the population of interest and must undergo thorough psychometric evaluation in the target population. The results of the psychometric evaluations of the current study, along with the patient interviews, support its application in vaccine and treatment trials. Moreover, the SIC may have potential application in assessing the impact of variant-driven or vaccination-mediated changes in COVID-19 disease severity or symptoms.

Our goal in developing the SIC was to include, as much as possible and to the best of our knowledge, all symptoms experienced by COVID-19 patients based on literature reviews, patient input, and expert opinion. Although the cross-sectional study was limited to US patients, analyses were also conducted on a subset of data from ENSEMBLE2, a multinational, phase 3, COVID-19 vaccine trial. This dataset supports the validity of the SIC to assess signs and symptoms of COVID-19 in a variety of regions and settings. Because a vaccine trial is not optimal to establish meaningful change thresholds, further studies will be needed to more definitively establish these thresholds. Furthermore, symptoms experienced by COVID-19 patients are heterogeneous, with variability among patients in both type and severity of symptoms. SARS-CoV-2 is also changing rapidly; for example, with the continuous emergence of new variants [3–5] (most recently Omicron XBB.1.5, as of February 2023), additional qualitative studies will be needed to assess how new variants affect the symptomatic experience of COVID-19. It is possible the patient experience of COVID-19 as captured in this analysis could evolve as the COVID-19 variant landscape continues to change. Although ENSEMBLE2 data present change over time, long-term symptoms and effects of COVID-19 also remain to be investigated. However, the additional evidence obtained from the exit interviews may support a potential role for the SIC in diagnosis and evaluation of post–acute sequelae of COVID-19.

## Conclusion

The results of the psychometric analyses and exit interviews from the cross-sectional study along with the longitudinal psychometric analyses from ENSEMBLE2 support use of the SIC in both treatment and vaccine clinical trials, pending future work to evaluate the responsiveness of the SIC.

## Electronic supplementary material

Below is the link to the electronic supplementary material.

Supplementary Appendix: Supplementary Tables and Figure

## Data Availability

The data sharing policy of Janssen Pharmaceutical Companies of Johnson & Johnson is available at https://www.janssen.com/clinical-trials/transparency. These data were made available by RTI Health Solutions for the current study and are not publicly available. Other researchers should contact https://www.rtihs.org/.

## List of Abbreviations

ANOVA: Analysis of variance
CDC: Centers for Disease Control and Prevention
COVID-19: Coronavirus disease 2019
GED: General Education Development
PCR: Polymerase chain reaction
PGIC: Patient Global Impression of Change
PGIS: Patient Global Impression of Severity
PRO: Patient-reported outcome
PROMIS-29: Patient-Reported Outcomes Measurement Information System-29
RT: Reverse transcription
SD: Standard deviation
SIC: Symptoms of Infection with Coronavirus-19
VAS: Visual analog scale
