# Supplementary material for: Psychometric evaluation of the Symptoms of Infection with Coronavirus-19 (SIC): results from a cross-sectional study and a phase 3 clinical trial
Source: J Patient Rep Outcomes. 2023 May 17;7:45. doi: 10.1186/s41687-023-00581-z (PMC10189706; doi:10.1186/s41687-023-00581-z)
Supplement: Supplementary file 1 — Supplementary Appendix: Supplementary Tables and Figure [file 41687_2023_581_MOESM1_ESM.docx]

# Supplementary Appendix

# Supplementary Tables

**Table S1. Signs and Symptoms Included in the SIC**

| **Please rate the severity of each symptom you experienced (in the last 24 hours)** | **Rating^a^** |
| --- | --- |
| C1 Feeling generally unwell | 0-10 |
| C2 Fatigue (tiredness) | 0-10 |
| M1 Physical weakness | 0-10 |
| R1 Cough | 0-10 |
| R2 Shortness of breath (difficulty breathing) | 0-10 |
| R3 Sore throat | 0-10 |
| R4 Nasal congestion (stuffy nose) | 0-10 |
| R5 Wheezing (whistling sound while breathing) | 0-10 |
| R6 Runny nose | 0-10 |
| R7 Sneezing | 0-10 |
| R8 Chest congestion (mucus in chest) | 0-10 |
| R9 Chest pain/pressure/tightness | 0-10 |
| M2 Muscle aches/pains | 0-10 |
| M3 Joint aches/pains | 0-10 |
| N1 Headache | 0-10 |
| N2 Feeling faint | 0-10 |
| N3 Problems thinking clearly | 0-10 |
| C3 Chills | 0-10 |
| C4 Skin rash | 0-10 |
| C5 Eye irritation/discharge | 0-10 |
| G1 Diarrhea | 0-10 |
| G2 Vomiting | 0-10 |
| G3 Nausea | 0-10 |
| G4 Abdominal/stomach pain | 0-10 |
| G5 Loss of appetite | 0-10 |
| C6 Fever, if yes, _____ degrees | Y/N |
| C7 Uncontrollable body shaking/shivering | Y/N |
| N4 Decreased sense of smell | Y/N |
| N5 Decreased sense of taste | Y/N |
| V1 Red or bruised feet or toes | Y/N |

SIC, Symptoms of Infection with Coronavirus-19; C, Constitutional; M, Musculoskeletal; R, Respiratory; N, Neurologic; G, Gastrointestinal; V, Vascular.

^a^0 to 10 ratings are defined as 0 (none) to 10 (worst possible). Y/N questions indicate yes or no for present or absent.

## **Table S2. Additional Survey Questions for Identifying Meaningful Reductions in Symptom Severity (Cross-sectional Study)**

| Symptom questions | - Which of the following symptoms would have to improve (become less severe) for you to return to your usual activities (your everyday activities prior to becoming sick with COVID-19)? |
| --- | --- |
|  | - Which of the following symptoms would need to be completely gone before you could return to your usual activities (your everyday activities prior to becoming sick with COVID-19)? |
| Access questions | - How easy or difficult was it for you to get your most recent test for COVID-19? |
|  | - Outside of your COVID-19 testing, did you seek medical care to treat your COVID-19 symptoms? |
|  | - How easy or difficult was it to access care for your COVID-19 symptoms? |
|  | - What barriers or obstacles to receiving care for your COVID-19 symptoms did you encounter? |

COVID-19, coronavirus disease 2019.

## **Table S3. Frequently Endorsed SIC Item-level Responses (ENSEMBLE2 Phase 3 Trial; N = 130^a^)**

|  | **Symptom frequency,**  **n (%; N = 130**^a^**)** | |
| --- | --- | --- |
| **SIC item** | No | Yes |
| Feeling unwell | 39 (30.0) | 91 (70.0) |
| Cough | 42 (32.3) | 88 (67.7) |
| Headache | 43 (33.1) | 87 (66.9) |
| Fatigue | 54 (41.5) | 76 (58.5) |
| Sore throat | 66 (50.8) | 64 (49.2) |

SIC, Symptoms of Infection with Coronavirus-19; COVID-19, coronavirus disease 2019; COVID-19, coronavirus disease 2019.

^a^Of 183 participants with PCR-confirmed moderate to severe/critical COVID-19 occurring from Days 15 to 56 post–primary vaccination, 130 completed the SIC.

## **Table S4. Descriptive Statistics for the PGIS and PGIC**

| **Patient Global Impression Item** | **n (%; N = 152)** | **Mean (SD)** |
| --- | --- | --- |
| **PGIS – Overall Sample** |  |  |
| 0 (No symptoms) | 22 (14.5) | NA |
| 1 (Mild) | 66 (43.4) | NA |
| 2 (Moderate) | 54 (35.5) | NA |
| 3 (Severe) | 10 (6.6) | NA |
| Age <65 years | 88 (57.9) | 1.24 (0.8) |
| Age ≥65 years | 64 (42.1) | 1.48 (0.8) |
| With no comorbidities | 79 (52.0) | 1.35 (0.8) |
| With comorbidities | 73 (48.0) | 1.33 (0.8) |
| **PGIC – Overall Sample** |  |  |
| 0 (Much better) | 56 (36.8) | NA |
| 1 (Moderately better) | 40 (26.3) | NA |
| 2 (A little better) | 22 (14.5) | NA |
| 3 (No change) | 13 (8.6) | NA |
| 4 (A little worse) | 11 (7.2) | NA |
| 5 (Moderately worse) | 9 (5.9) | NA |
| 6 (Much worse) | 1 (0.7) | NA |
| Age <65 years | 88 (57.9) | 1.25 (1.6) |
| Age ≥65 years | 64 (42.1) | 1.69 (1.4) |
| With no comorbidities | 79 (52.0) | 1.37 (1.5) |
| With comorbidities | 73 (48.0) | 1.51 (1.6) |

PGIS, Patient Global Impression of Severity; PGIC, Patient Global Impression of Change.

## **Table S5. Test-retest Reliability Among Stable Patients (ENSEMBLE2 Phase 3 Trial)**

| **PGIS response of “no symptom” or “mild” at Day 1 and Day 2, N = 74** | **ICC (95% CI)** |
| --- | --- |
| SIC composite score |  |
| Constitutional | 0.54 (0.39-0.70) |
| Gastrointestinal | 0.67 (0.53-0.78) |
| Musculoskeletal | 0.60 (0.44-0.73) |
| Neurologic | 0.50 (0.34-0.66) |
| Respiratory | 0.73 (0.61-0.82) |
| Lower respiratory | 0.67 (0.54-0.78) |
| Upper respiratory | 0.72 (0.60-0.81) |
| Sensory | 0.75 (0.63-0.83) |
| **Same PGIS response at Day 1 and Day 2, N = 80** | **ICC (95% CI)** |
| SIC composite score |  |
| Constitutional | 0.61 (0.46-0.73) |
| Gastrointestinal | 0.72 (0.61-0.81) |
| Musculoskeletal | 0.70 (0.57-0.80) |
| Neurologic | 0.52 (0.37-0.67) |
| Respiratory | 0.70 (0.58-0.80) |
| Lower respiratory | 0.64 (0.51-0.76) |
| Upper respiratory | 0.70 (0.58-0.80) |
| Sensory | 0.80 (0.70-0.86) |

PGIS, Patient Global Impression of Severity; ICC, intraclass correlation coefficient; CI, confidence interval.
ICCs of 0.2 to 0.4 indicate fair agreement, 0.41 to 0.60 indicate moderate agreement, 0.61 to 0.80 indicate strong agreement, and 0.81 to 1.00 indicate near complete agreement.

## **Table S6. Mean Change in SIC Scores in Patients with PGIS Improvement^a^ by Day (ENSEMBLE2 Trial)**

| **SIC composite score** | **Day 1 to Day 2 (N = 20)** | **Day 1 to Day 3 (N = 23)** | **Day 1 to Day 5 (N = 28)** |
| --- | --- | --- | --- |
| Constitutional | −1.39 | −1.53 | −1.65 |
| Gastrointestinal | −0.36 | −0.21 | −0.64 |
| Musculoskeletal | −1.02 | −1.77 | −2.11 |
| Neurologic | −1.18 | −1.36 | −1.04 |
| Respiratory | −1.09 | −0.94 | −0.98 |
| Lower respiratory | −0.89 | −0.74 | −0.59 |
| Upper respiratory | −1.35 | −1.20 | −1.47 |
| Sensory | 0.00 | −0.22 | −0.36 |

SIC, Symptoms of Infection with Coronavirus-19; PGIS, Patient Global Impression of Severity.

^a^Improvement is defined as a 1- or 2-point change in PGIS score.

## **Table S7. Representative Quotations From Exit Interviews**

| **Participant no.** | **Comments** |
| --- | --- |
| 17 | I didn’t pass away or anything. I’m still here, so that’s good. Something like a 7. Because I did have to go to the hospital and be put on a ventilator. That’s bad. But like I said, it was nothing fatal or deathly, and I recovered just fine. |
| 20 | I was going to say loss of smell. At first, that was the most bothersome, but then it was the headache. So, I would definitely say the headaches; that just completely immobilized me. |
| 11 | The fever was very severe, and the shortness of breath, that was scaring the hell out of me. Because I was looking at, if I can't breathe, oh, shoot, this thing is taking me right now… |
| 3 | [Which symptoms need to be completely gone?] I would say the headache. The stuffiness I could live with... if I was okay to go to work, but I was so stuffy, I [couldn’t] do it. It would suck [because] a mask on top of your face while you’re [stuffy]. That feels like, “Oh, my gosh. I can’t breathe.” I guess if I had to go to work, I wouldn’t want to go to work that way, and the headache just makes you not want to get up. I for sure would have wanted that gone. |
| 18 | The difficulty breathing [needs to be gone]. Or just improved greatly. It would just be because I’m a single mom, so I do everything. So, if I’m sick, my son really can’t do much for himself like that. So, if I can’t breathe and make it to the kitchen and cook dinner, I’m really not much of a help right there, unfortunately. |
| 17 | [Could she have answered the survey while most ill?] Yeah. If I was awake for a period of more than 2 hours, then yes…. When I woke up from every time I slept, that was kind of the worst grogginess that I had. Because it took me 30 minutes to actually wake up and kind of realize what’s going on… they would ask me when I wasn’t sleeping, obviously. But yeah, they would ask me or they’d be like, “Hold up fingers. How do you feel?” |
| 5 | [Regarding taking the survey while ill] If he [spouse] read the questions and kind of took his time, I probably could, but the clarity would probably have been a little off or diminished, because I kind of… I call it COVID brain. I just was not thinking correctly. |

COVID, coronavirus disease.

# Supplementary Figure

## **Figure S1. SIC development schematic diagram.**


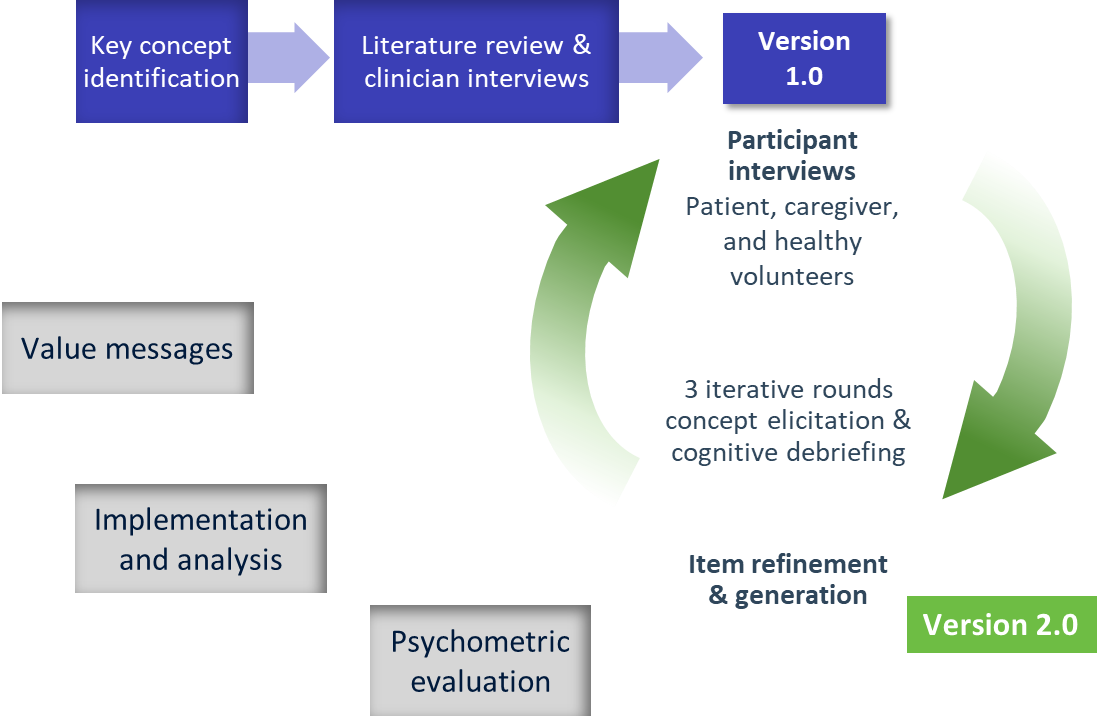


SIC, Symptoms of Infection with Coronavirus-19.
